# Xinfeng capsule attenuates ankylosing spondylitis by downregulating YTHDC1-mediated m^6^A modification of LINC01579 and suppressing IL-17/NF-κB signaling

**DOI:** 10.3389/fimmu.2026.1762062

**Published:** 2026-04-16

**Authors:** Xiang Ding, Jian Liu, Xiaolu Chen, Xian-Heng Zhang, Yanyan Fang

**Affiliations:** 1 Department of Rheumatology and Immunology, First Affiliated Hospital of Anhui University of Traditional Chinese Medicine, Hefei, Anhui, China; 2 Institute of Rheumatology, Anhui University of Chinese Medicine, Hefei, Anhui, China; 3 First Clinical Medical College, Anhui University of Traditional Chinese Medicine, Hefei, Anhui, China

**Keywords:** ankylosing spondylitis, epigenetics, immunoinflammation, methylation, Xinfeng capsule

## Abstract

**Background:**

Ankylosing spondylitis (AS) is a chronic inflammatory disorder predominantly affecting the sacroiliac and spinal joints. Emerging evidence indicates that N^6^-methyladenosine (m^6^A) RNA modification plays a critical role in inflammatory regulation. Xinfeng Capsule (XFC), a Traditional Chinese Medicine formula (Xin’an medicine), has demonstrated clinical efficacy in alleviating AS-associated inflammation. However, whether XFC modulates AS pathology through m^6^A-dependent epigenetic mechanisms remains unclear.

**Objective:**

This study aimed to investigate whether XFC mitigates AS inflammation by regulating the IL-17/NF-κB pathway via m^6^A modification.

**Methods:**

Core therapeutic targets were identified by integrating network pharmacology with RNA-sequencing data. The direct interaction between YTHDC1 and LINC01579 was validated using RNA pull-down and dual-luciferase reporter assays. To identify the functional m6A sites, site-directed mutagenesis of two putative m6A motifs (MUT1 and MUT2) in LINC01579 was performed, and the effects of YTHDC1 knockdown were assessed. An *in vitro* co-culture model comprising AS patient-derived fibroblast-like synoviocytes (FLS) and peripheral blood mononuclear cells (PBMC) was established, alongside an *in vivo* proteoglycan-induced arthritis (PGIA) mouse model. To verify the underlying mechanism, gain- and loss-of-function experiments were conducted using overexpression plasmids and small interfering RNAs (siRNAs) targeting LINC01579 and YTHDC1. m^6^A levels were quantified by MeRIP-qPCR. Inflammation and pathway activation were assessed via immunofluorescence, Western blot, and enzyme-linked immunosorbent assay (ELISA). RNA stability was evaluated using actinomycin D assays, while cell migration was measured by scratch assays. Bone destruction was analyzed using micro-CT and histological staining. Additionally, an IL-17 pathway inhibitor (AIN457) and agonist (SR0987) were employed to validate pathway involvement.

**Results:**

Bioinformatics and network pharmacology analysis identified LINC01579 as a core gene in AS inflammation and YTHDC1 as an upstream regulator of LINC01579 m^6^A modification. RNA pull-down assays confirmed the direct binding of YTHDC1 to LINC01579.Functional studies revealed that mutation of the MUT1 site abolished the regulatory effects of YTHDC1 knockdown on inflammatory cytokines (IL-17, IL-6, TNF-α) and LINC01579 expression. XFC was predicted to inhibit inflammation via the IL-17/NF-κB pathway. *In vitro*, LINC01579 knockdown significantly enhanced the release of inflammatory mediators and activated IL-17/NF-κB signaling. Conversely, YTHDC1 overexpression increased LINC01579 m^6^A levels, leading to its downregulation. XFC treatment effectively reduced YTHDC1 expression and LINC01579 m^6^A modification, thereby restoring LINC01579 levels and suppressing IL-17/NF-κB activation. *In vivo*, XFC ameliorated joint inflammation, bone erosion, and joint space narrowing in PGIA mice.

**Conclusion:**

XFC alleviates AS progression by inhibiting YTHDC1-mediated m6A modification of LINC01579, which prevents its degradation and subsequently dampens IL-17/NF-κB pathway activation. These findings highlight a potential epigenetic mechanism underlying the therapeutic effects of XFC in AS.

## Graphical Abstract

**Figure:**
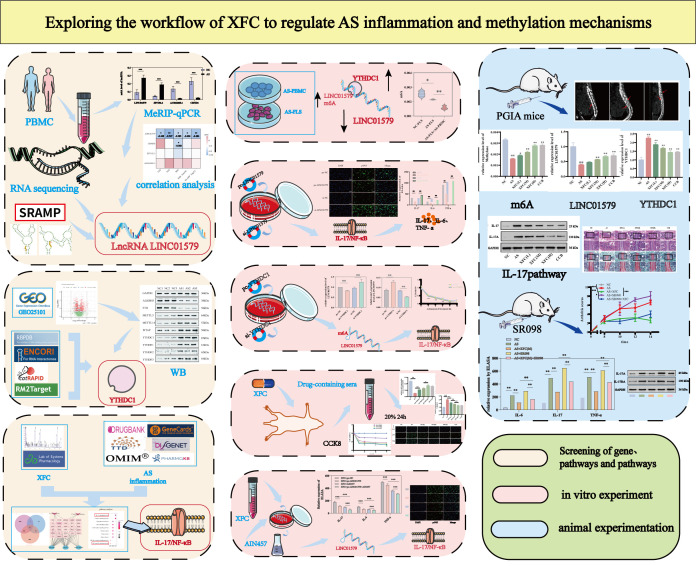

## Introduction

Ankylosing spondylitis (AS) is a highly heritable spondyloarthritis characterized primarily by inflammatory chronic low back pain, predominantly involving the sacroiliac and spinal joints (1). The presence of Human leukocyte antigen B27 (HLA-B27) is observed in ~90% of patients with AS (2), compared with <9% in the general population (3), representing one of the strongest known associations between a genetic variant and a complex disease. Although the genetic underpinnings of AS have been intensively investigated (4), effective therapies remain limited, reflecting both the complexity of the disease and an incomplete understanding of its pathogenesis (5). In recent years, attention has shifted toward epigenetic regulation, particularly RNA methylation, in AS. While advances in sequencing have enabled the cataloguing of differentially methylated genes, evidence suggests that the heritability of methylation levels may be lower than previously anticipated (6).

N^6^-methyladenosine (m^6^A) is the most prevalent epigenetic modification of eukaryotic RNA, primarily mediated by m^6^A regulators, including methyltransferases, demethylases, and binding proteins that coordinate m^6^A recognition and function (7). These methylation-related enzymes participate in various pathological processes in AS, such as inflammation, autophagy, and osteogenic differentiation. Despite their lack of coding capacity, long noncoding RNAs (lncRNAs) harboring m^6^A modifications influence disease initiation and progression mainly by modulating interactions between lncRNAs and other RNAs or proteins(Y. 8, 9). Although these modifications do not alter the fundamental principles of complementary base pairing, they impact RNA metabolism—such as splicing, localization, and translation—thereby affecting transcript fate (10). In this study, we identified differentially expressed lncRNAs in peripheral blood mononuclear cells (PBMCs) from patients with AS. Among them, long intergenic non-protein coding RNA 1579 (LINC01579) was significantly downregulated and showed strong correlations with clinical indicators and patient-reported outcomes (11). Interestingly, this expression pattern contrasts with that observed in renal cell carcinoma, suggesting potential disease specificity. However, to date, no studies have investigated whether LINC01579 exerts any molecular function in the context of AS.

Although modern treatments for AS have become increasingly diversified, a subset of patients remains unresponsive to existing therapeutic options (12). Traditional Chinese medicine (TCM) has a long-standing history in AS management. Xinfeng Capsule (XFC), a TCM formulation composed of *Astragali Radix* (*Astragalus membranaceus*), *Coicis Semen* (*Coix lacryma-jobi* var. *ma-yuen*), *Tripterygium wilfordii* Hook. F., and *Scolopendra subspinipes mutilans* in a ratio of 20:20:10:1, has been used clinically for over 20 years. It is widely applied in the treatment of immune-related disorders and has demonstrated favorable safety and efficacy profiles through fingerprinting analysis and long-term clinical data (13). XFC has shown significant therapeutic potential in alleviating AS-associated immune inflammation (14), bone metabolic imbalance (15), and hypercoagulability (16). Mechanistically, studies have indicated that XFC may exert anti-inflammatory effects via modulation of the NF-κB signaling pathway (17), thus contributing to its efficacy in AS. Despite advances in molecular therapies for AS, the underlying mechanisms remain incompletely understood. Targeted molecular interventions that regulate disease-associated gene expression hold promise for slowing disease progression, but their clinical application warrants further investigation.

In this study, we explored whether XFC mitigates AS-associated immune inflammation by inhibiting YTHDC1-mediated m^6^A modification of LINC01579. Our findings provide novel mechanistic insight and support the therapeutic potential of XFC in AS treatment.

## Materials and methods

### Clinical patients

Comprehensive clinical data from 30 patients diagnosed with AS were retrieved from the electronic medical records of the Rheumatology Department, First Affiliated Hospital of Anhui University of Traditional Chinese Medicine. The study protocol was approved by the hospital’s Research Ethics Committee (Approval No. 2024AH-92) and registered with the International Traditional Medicine Clinical Trial Registry (ITMCTR) on September 20, 2024 (Registration No. ITMCTR2024000692). The assessment included a range of clinical parameters, including patient-reported outcomes such as the Self-Rating Anxiety Scale (SAS), Self-Rating Depression Scale (SDS), and Visual Analogue Scale (VAS), as well as inflammatory markers including erythrocyte sedimentation rate (ESR) and high-sensitivity C-reactive protein (hs-CRP). Peripheral blood samples were collected at admission for cytokine profiling, gene expression analysis, and the isolation of PBMCs.

### Animal model

The proteoglycan-induced arthritis (PGIA) mouse model, which mimics inflammatory pathology observed in AS, was established using human articular cartilage-derived proteoglycans. A total of 61 female BALB/c mice (8 weeks old, 26 ± 2.4 g) were purchased from Viton Lever Laboratory Animal Technology Co., Ltd. (Animal Certificate No. 20241021Abzz0619000342). All procedures were approved by the Laboratory Animal Ethics Committee of Anhui University of Traditional Chinese Medicine (Approval No. AHUCM-rats-2023020). PGIA was induced via intraperitoneal injection of 100 μg proteoglycan (PG; Sigma, St. Louis, MO, USA) emulsified with 2 mg dimethyl dioctadecylammonium (DDA; Sigma) on days 0, 21, and 42. Control mice received an equivalent volume of sterile water at the same time points (18).

Thirty-six mice were randomly assigned to six groups (n = 6 per group): normal, AS model, XFC low-dose, XFC medium-dose, XFC high-dose, and celecoxib group. Oral gavage administration began seven weeks after PG induction. The XFC doses (based on prior studies) were 0.72, 1.44, and 2.88 g/kg for low, medium, and high-dose groups, respectively. Celecoxib was administered at 0.3 mg/kg. Both the normal and AS groups received saline by gavage at a volume of 10 mL/kg. To investigate the mechanism involving the IL-17 pathway, an additional 25 mice were randomized into five groups (n = 5 per group): negative control (NC), AS, AS+XFC, AS+IL-17 agonist (SR0987), and AS+IL-17 agonist (SR0987)+XFC. During the 7-week modeling phase, SR0987 was administered intraperitoneally every other day (19). Mice in the SR0987 and SR0987+XFC groups received 0.1 mg SR0987 dissolved in 0.25 mL dimethyl sulfoxide (DMSO) per mouse. Following the 7-week modeling period, mice in the XFC and SR0987+XFC groups were treated with XFC (1.44 g/kg) via oral gavage. On the final day, 1 hour after gavage, all mice were anesthetized with 1% sodium pentobarbital (30 mg/kg, i.p.), and samples including abdominal aortic blood, liver, kidney, and spinal joint tissues were collected for analysis (20).

### Preparation of XFC-containing serum

Thirty specific pathogen-free (SPF) grade male Sprague-Dawley (SD) rats (220 ± 20 g) were randomly assigned using a numerical table to either the XFC-containing serum group (n = 20) or the normal serum control group (n = 10). Rats in the intervention group received XFC suspension via oral gavage at a dose of 6.48 g/kg/day, while control rats were administered an equal volume of saline. After seven consecutive days of treatment, animals were anesthetized 1 hour after the final dose using intraperitoneal sodium pentobarbital (50 mg/kg). Blood was then collected via the abdominal aorta, and drug-containing serum was prepared following a standardized protocol established by the research group (17).

### Mouse arthritis scoring and micro-computed tomography

Arthritis scoring was performed weekly by two independent investigators blinded to the treatment groups. The severity of arthritis was evaluated using a standardized visual scoring system based on the criteria described by Ishikawa et al. (21): 0, normal; 1, slight swelling and/or redness of a single digit; 2, moderate swelling and erythema of a single digit; 3, marked swelling and erythema of the paw, including all joints and the ankle; and 4, swelling involving the entire paw. The cumulative arthritis score was calculated by summing the scores of all four limbs, with a maximum total of 16 per mouse. At week 14, all mice underwent micro-CT scanning using the SkyScan 1176 system (Bruker, Germany) to assess bone structural changes. Following imaging, all animals were sacrificed, and spinal joint tissues were collected. Histopathological changes were evaluated using hematoxylin and eosin (HE) staining to assess inflammation and Safranin O-fast green staining to visualize cartilage integrity and proteoglycan loss.

### Origin and culture of AS-fibroblast-like synoviocytes and AS-PBMCs

Human primary FLSs (Catalog No. RAB-iCell-s004) and AS-derived FLSs (AS-FLSs; Catalog No. JDBG200752), isolated from sacroiliac joint tissue, were obtained from Subikang iCell Co. (Shanghai, China). AS-PBMCs were isolated from venous blood samples collected from AS patients with ethics approval (Approval No. 2015-AH20). Cells were cultured in RPMI-1640 medium (Subikang Biotechnology, Shanghai) at 37 °C in a humidified atmosphere containing 5% CO_2_.

### Co-culture model of AS-PBMCs and AS-FLSs

AS-PBMCs and AS-FLSs were co-cultured in a Transwell system at a ratio of 3:1. PBMCs were seeded in the upper chamber, while FLSs were plated in the lower chamber. The chambers were incubated at 37 °C with 5% CO_2_ for 24 hours. Once FLS confluence reached approximately 70-90%, the Transwell inserts were removed, and the AS-FLSs were collected from the lower chamber for subsequent experiments.

### RNA extraction, reverse transcription-quantitative PCR, and stability assessment

To assess RNA stability, cells were treated with actinomycin D (W10655, Shanghai Yuanye Biotechnology) for 0, 2, 4, 6, 8, and 10 hours. Total RNA was extracted from synovial tissue using 1 mL of TRIzol reagent (Thermo Fisher Scientific) following the manufacturer’s protocol. RNA concentration and purity were determined using a spectrophotometer. cDNA synthesis was carried out using the TIANScript RT Kit (Tiangen, China) according to the manufacturer’s instructions. Quantitative PCR was performed using SYBR Green Master Mix on a real-time PCR system with gene-specific primers targeting both the genes of interest and internal reference genes. The primer sequences used in this study are listed in **Table 1**.

**Table 1:** Primers for target genes.

Gene	Amplicon size (bp)	Forward primer (5’→3’)	Reverse primer (5’→3’)
β-actin	96	CCCTGGAGAAGAGCTACGAG	GGAAGGAAGGCTGGAAGAGT
U6	94	CTCGCTTCGGCAGCACA	AACGCTTCACGAATTTGCGT
LINC01579	197	GTCTGTAAACGGTTCTCCAGGG	CCACATAAGCAGGTGTGGCT
YTHDC1	173	GAAGCAGGCTCCATTTTAGC	TAACACTCAGCCTTCTCGAC

### Enzyme-linked immunosorbent assay

Cell culture supernatants and mouse serum samples were collected from AS, NC, and PGIA model groups (Ethics Approval No. 2023AH-52). The levels of tumor necrosis factor-α (TNF-α, JYM0110Hu), interleukin-17A (IL-17A; JYM1702Hu), and IL-6 (JYM0140Hu) were quantified using commercial ELISA kits, following the manufacturer’s instructions.

### Colony formation assay

Cells were digested with trypsin and seeded into culture dishes at a density of 1,000 cells per dish in 10 mL of medium per dish. After incubation for 2–3 weeks, colonies were fixed with 4% paraformaldehyde and stained with crystal violet solution for 15 minutes. Colonies consisting of more than 10 cells were counted under a microscope(Bai et al., 2022).

### Nucleoplasmic separation assay

For nuclear and cytoplasmic RNA isolation, total RNA was extracted using a nucleoplasmic separation kit (AmyJet Scientific, Cat. No. 21000, China) in accordance with the manufacturer’s instructions. The relative distribution of RNA between the nucleus and cytoplasm was assessed via RT-qPCR using the comparative ΔCt method.

### Western blot

Cells were lysed using radioimmunoprecipitation assay (RIPA) buffer and centrifuged at 10,000 × g for 10 minutes. The supernatant containing total protein was collected and mixed with 5× SDS loading buffer at a 1:4 volume ratio. Samples were denatured by boiling and subjected to sodium dodecyl sulfate-polyacrylamide gel electrophoresis (SDS-PAGE) using precast gels. Proteins were transferred onto a polyvinylidene difluoride (PVDF) membrane, which was pre-activated in methanol for 2 minutes and equilibrated in transfer buffer for 5 minutes. Following transfer, membranes were rinsed with TBST and incubated overnight at 4 °C with primary antibodies, followed by incubation with horseradish peroxidase (HRP)-conjugated secondary antibodies (1:20,000 dilution) for 1 hour at room temperature. After three washes with TBST, protein bands were visualized using enhanced chemiluminescence detection.

### MeRIP-qPCR

Total RNA was extracted from co-cultured FLSs, and an m^6^A antibody pre-bound to immunomagnetic beads was added to the RNA samples. The RNA-antibody complexes were enriched using a magnetic separation rack, followed by protease digestion to release the bound RNA. Primers specific for LINC01579 were designed, and subsequent quantification was performed by qPCR to evaluate the m^6^A modification level of LINC01579 after co-culture.

### Immunofluorescence

The IF protocol was performed according to the previously established method by our research group (22). Briefly, cells cultured on plates were fixed, permeabilized, blocked, and sequentially incubated with primary and fluorophore-conjugated secondary antibodies. After staining, the samples were mounted with an anti-fade mounting medium, and fluorescence signals were visualized using a digital slide scanner.

### Histopathologic testing and evaluation

Spinal joint tissues from PGIA mice were harvested, sectioned, and subjected to HE staining as well as Safranin O/Fast Green staining to assess tissue morphology and cartilage integrity.

### Target gene overlap and functional enrichment analysis

To identify potential therapeutic targets of XFC, core bioactive components derived from the XFC fingerprint and AS-related targets were overlapped using Venn diagram software (https://bioinformatics.com.cn/static/others/jvenn/example.html). The resulting intersecting genes were regarded as candidate targets of XFC in AS. These targets were further analyzed through Gene Ontology (GO) and Kyoto Encyclopedia of Genes and Genomes (KEGG) enrichment to identify relevant biological processes and signaling pathways involved in the potential mechanisms of action of XFC.

### Protein-protein interaction network

PPI networks were constructed to explore the interactions among target genes within the enriched KEGG pathways. The PPI network was visualized using Cytoscape software (v3.6.1), and hub genes were identified based on node degree centrality.

### GO and KEGG analysis

The R package clusterProfiler was used to perform functional enrichment analyses. Potential target genes were imported into R software and converted to Entrez gene IDs based on gene symbols. GO and KEGG analyses were performed using a *p*-value cutoff of 0.05, and the results were visualized using R.

### Cell counting kit-8

Cell viability was assessed using the CCK-8 kit (BS350B, BioShari, China). Synovial cells were suspended at a concentration of 5-10×10^4^/mL and seeded into 96-well plates. At 0, 12, 24, 48, and 72 hours, 10 μL of CCK-8 solution was added to each well. After 3 hours of incubation, absorbance was measured at 450 nm using a microplate reader. Blank wells containing medium only were included for background correction.

### Bioinformatics analysis of differentially expressed genes

The AS gene expression dataset GSE25101 was downloaded from the Gene Expression Omnibus (GEO), comprising whole-blood samples from 16 patients with active AS and 16 age- and sex-matched healthy controls. The control group was used as the baseline, and differential expression analysis was conducted by integrating the GSE25101 dataset with RNA-seq data generated by our group (23) using the limma package in R software (24). Genes with |log_2_(Fold Change [FC])| > 2 and *p* < 0.05 were considered significantly differentially expressed.

### Dual luciferase reporter gene assay

AS-FLSs co-cultured with AS-PBMCs were seeded into 96-well plates at approximately 50-70% confluency. For each well, 0.16 μg of target plasmid encoding either wild-type or mutant hsa-LINC01579 (WT, MUT1, MUT2, or MUT1+MUT2) and 0.16 μg of hsa-YTHDC1 plasmid (NC or siRNA) were mixed with 5 μL of DMEM (Solution A). Separately, 0.3 μL of transfection reagent was diluted in 5 μL of DMEM (Solution B). Solutions A and B were combined and incubated for 20 minutes at room temperature. After replacing the culture medium, the transfection mixture was added to the cells. Six hours post-transfection, the medium was replaced again, and cells were harvested 48 hours later for luciferase activity measurement. Firefly and Renilla luciferase activities were quantified using the Dual-Luciferase^®^ Reporter Assay System (Promega) according to the manufacturer’s instructions. Renilla luciferase activity was used for normalization to evaluate relative reporter gene expression.

### RNA pull-down and qPCR assay

To investigate the interaction between LINC01579 and YTHDC1, an RNA pull-down assay was performed using a commercially available kit (Guangdong Huijun, China). Approximately 2-4 × 10^7^ cells were collected and lysed in Lysis Buffer supplemented with protease and RNase inhibitors on ice. The cell lysates were divided into two equal portions for the experimental and control groups. Meanwhile, streptavidin magnetic beads were pre-blocked and incubated with biotin-labeled specific LINC01579 probes (experimental group) or negative control probes (control group) for 30 min at room temperature. The probe-coated beads were then washed and incubated with the cell lysates overnight at 4 °C with rotation. After incubation, the beads were washed thoroughly with Wash Buffer to remove non-specific binders. The bound RNA-protein complexes were eluted, and total RNA was extracted from the pull-down samples using TRIzol reagent (Life Technologies, USA). RNA was reverse transcribed into cDNA using the PrimeScript™ RT reagent Kit with gDNA Eraser (TaKaRa, Japan) following the manufacturer’s protocol. Quantitative real-time PCR (qPCR) was performed using Novostart SYBR qPCR SuperMix Plus (novoprotein, China) on a PIKOREAL 96 system (Thermo Scientific, USA). The specific primers for YTHDC1 were as follows: forward, 5’-TCAGGCTGGAGAATAACGAC-3’; reverse, 5’-AGGTTGTGTGCTTGTAGGAA-3’. The relative enrichment of YTHDC1 was calculated using the 2^(-ΔΔCt) method and normalized to the control group.

### Statistical analysis

Statistical analyses were performed using GraphPad Prism (version 10.1.2) and SPSS (version 2023). Data normality was assessed using the Shapiro–Wilk test. Normally distributed data are presented as mean ± standard deviation (SD), whereas non-normally distributed data are expressed as median with interquartile range [M (P25, P75)]. Comparisons between two independent groups were conducted using two-tailed Student’s t-tests or the Wilcoxon rank-sum test, as appropriate. Comparisons among multiple groups were performed using one-way analysis of variance (ANOVA) followed by Bonferroni correction for multiple comparisons. Sample size estimation was performed using G*Power software. For the human cohort, an *a priori* power analysis (α = 0.05, power = 0.80) indicated that a minimum of 26 subjects was required, and therefore 30 participants were included in the study. For animal experiments, group sizes of 5–6 mice were determined based on ethical considerations and experimental feasibility; a *post hoc* power analysis demonstrated that the primary outcome measures achieved a statistical power exceeding 80%. All statistical tests were two-sided, and a *p* value < 0.05 was considered statistically significant.

## Results

### LINC01579 is a core inflammation-related lncRNA in AS and contains m^6^A methylation sites

**Figure 1:**
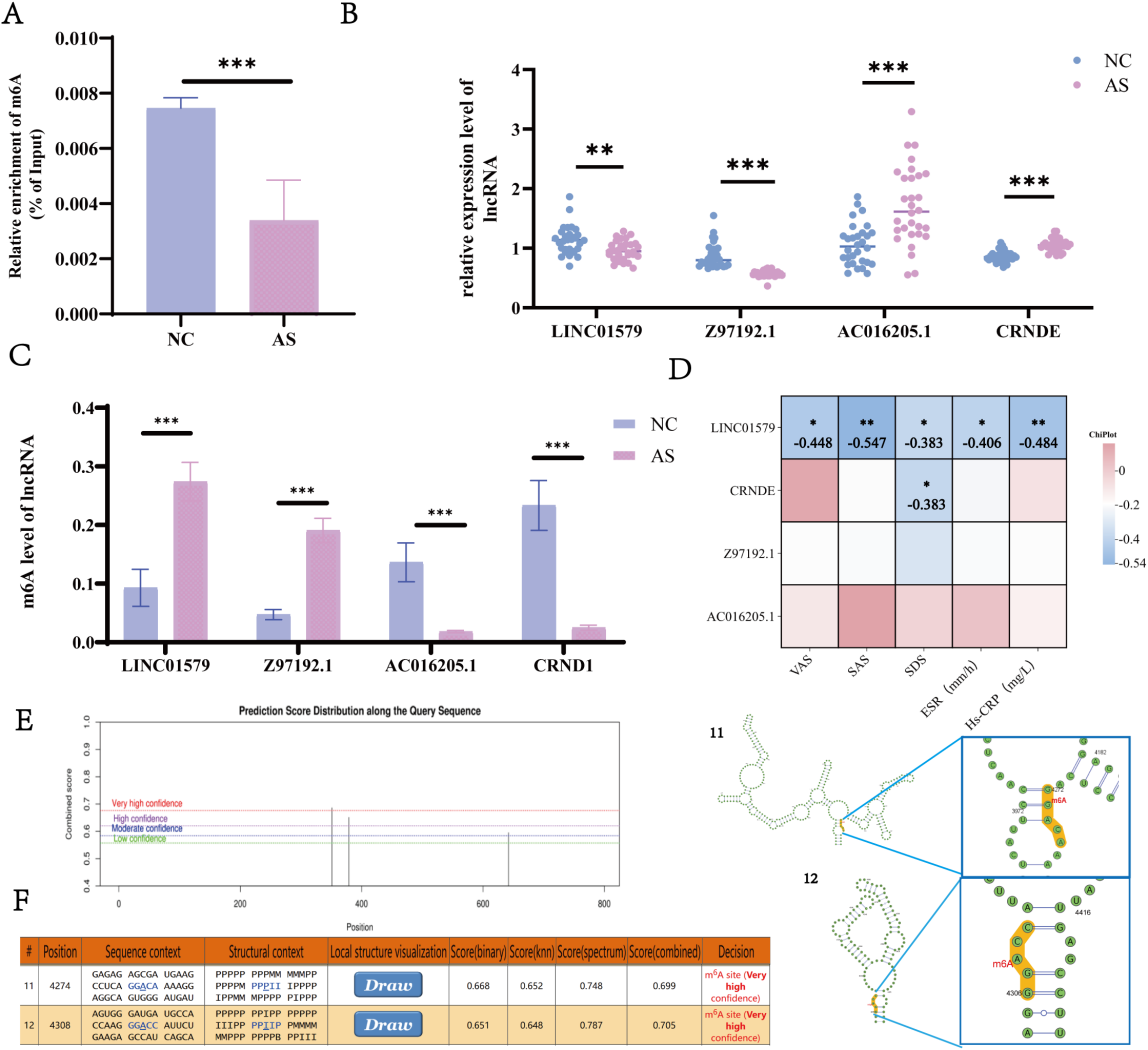
AS immunoinflammatory core gene screening. **(A)** Total m6A of AS and NC groups; **(B)** qRT-PCR to detect the expression of LINC01579, Z97192.1, AC016205.1, and CRNDE; **(C)** MeRIP-qPCR to detect LINC01579, Z97192.1, AC016205.1, and CRNDE expression; **(D)** Correlation analysis of LINC01579, Z97192.1, AC016205.1, and CRNDE with patient’s feelings and inflammation indexes; **(E)** SRAMP software predicted LINC01579 methylation sites; **(F)** High-confidence methylation sites (position 11,12). All experiments were repeated three times. **p < 0.01; ***p < 0.001.

MeRIP-qPCR analysis revealed that m^6^A levels in the peripheral blood of AS patients were significantly lower compared to healthy controls (*p* < 0.05) (**Figure 1A**). High-throughput whole-transcriptome sequencing conducted by our team identified four differentially expressed lncRNAs in AS patients (*p* < 0.05, FC > 2): LINC01579, Z97192.1, AC016205.1, and CRNDE. Among these, LINC01579 and Z97192.1 were significantly downregulated (*p* < 0.05), whereas AC016205.1 and CRNDE were upregulated (*p* < 0.05), as confirmed by RT-qPCR (**Figure 1B**). Their corresponding m^6^A methylation levels exhibited an opposite trend (*p* < 0.05) (**Figure 1C**). Clinical correlation analysis indicated that LINC01579 expression was negatively correlated with multiple clinical indices, including SAS, SDS, VAS, ESR, and hs-CRP (*p* < 0.05). Additionally, CRNDE expression was negatively correlated with SDS (*p* < 0.05) (**Figure 1D**). These findings suggest that LINC01579 may play a critical role in both inflammatory processes and patient symptom perception in AS. To determine whether LINC01579 contains m^6^A modification sites, the SRAMP prediction tool (http://www.cuilab.cn/sramp/) was employed (**Figure 1E**), which identified highly probable m^6^A sites, with the strongest predicted sites located at nucleotide positions 11 and 12 (**Figure 1F**). Based on the above findings, LINC01579 was identified as a core lncRNA involved in the pathogenesis of AS.

### LINC01579 may be regulated by the methyltransferase YTHDC1

**Figure 2:**
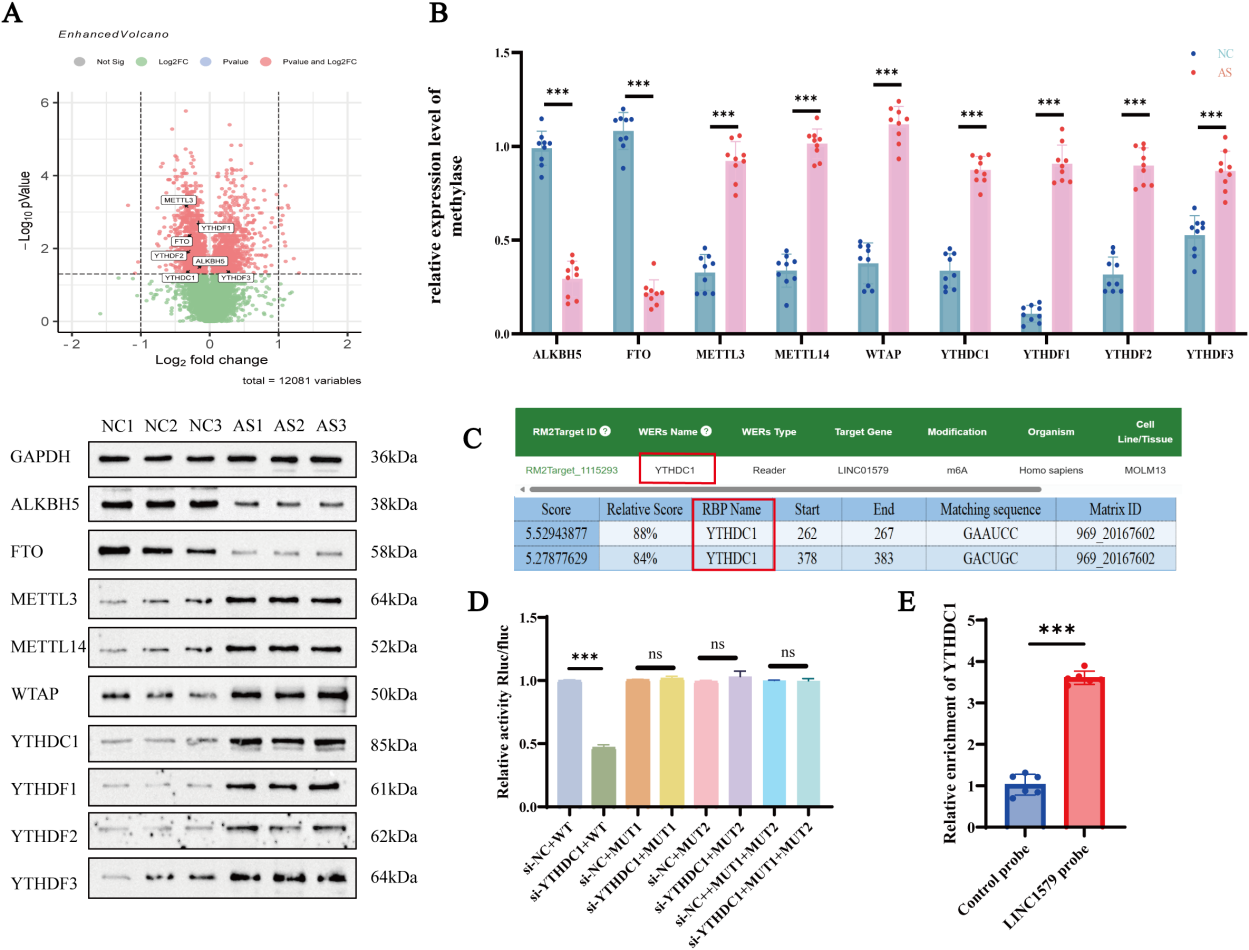
LINC01579 target methylase and reading protein screen. **(A)** GSE25101 database analysis of differential methylase heatmaps; **(B)** WB assay for methylase expression; **(C)** RM2Target and catRAPID online sites for prediction of LINC01579-binding methylases; **(D)** Dual luciferase assay; **(E)** Validation of the interaction between LINC01579 and YTHDC1 by RNA Pull-down assay. All experiments were repeated three times. **p < 0.01; ***p < 0.001.

Based on the GSE25101 dataset, differentially expressed m^6^A-related enzymes were screened using the criteria of *p* < 0.05 and fold change > 0.5 (**Figure 2A**). Combined with previous literature on m^6^A regulatory factors involved in AS, a total of eight candidate enzymes were identified: ALKBH5, FTO, YTHDC1, METTL14, WTAP, YTHDF1, YTHDF2, and YTHDF3. WB analysis of peripheral blood samples from healthy controls (NC, n = 8) and AS patients (n = 8) revealed that the protein levels of ALKBH5 and FTO were significantly decreased, whereas YTHDC1, METTL14, WTAP, YTHDF1, YTHDF2, and YTHDF3 were significantly upregulated in AS patients (*p* < 0.001) (**Figure 2B**). To identify potential upstream m^6^A regulators of LINC01579, four databases, RBPDB, ENCORI, catRAPID, and RM2Target, were used for predictive analysis. The results consistently indicated that YTHDC1 may directly bind to LINC01579 (**Figure 2C**). Dual-luciferase reporter assays further confirmed this interaction. Compared with the si-NC group, luciferase activity was significantly reduced when si-YTHDC1 was transfected with the WT construct. In contrast, no significant differences were observed in luciferase activity between the WT and mutant constructs (MUT1, MUT2, or MUT1 + 2) following YTHDC1 knockdown (**Figure 2D**). These findings suggest that YTHDC1 may regulate the m^6^A modification of LINC01579, particularly at nucleotide positions 351 and 379. To investigate the interaction between LINC01579 and YTHDC1, an RNA pull-down assay was performed followed by qPCR to detect the enrichment of YTHDC1. The results showed that the LINC01579 probe significantly enriched YTHDC1 compared with the negative control probe (**Figure 2E**). This result indicates that YTHDC1 is a binding target of LINC01579.

### Network pharmacology analysis of XFC in regulating core immunoinflammatory targets and pathways in AS

**Figure 3:**
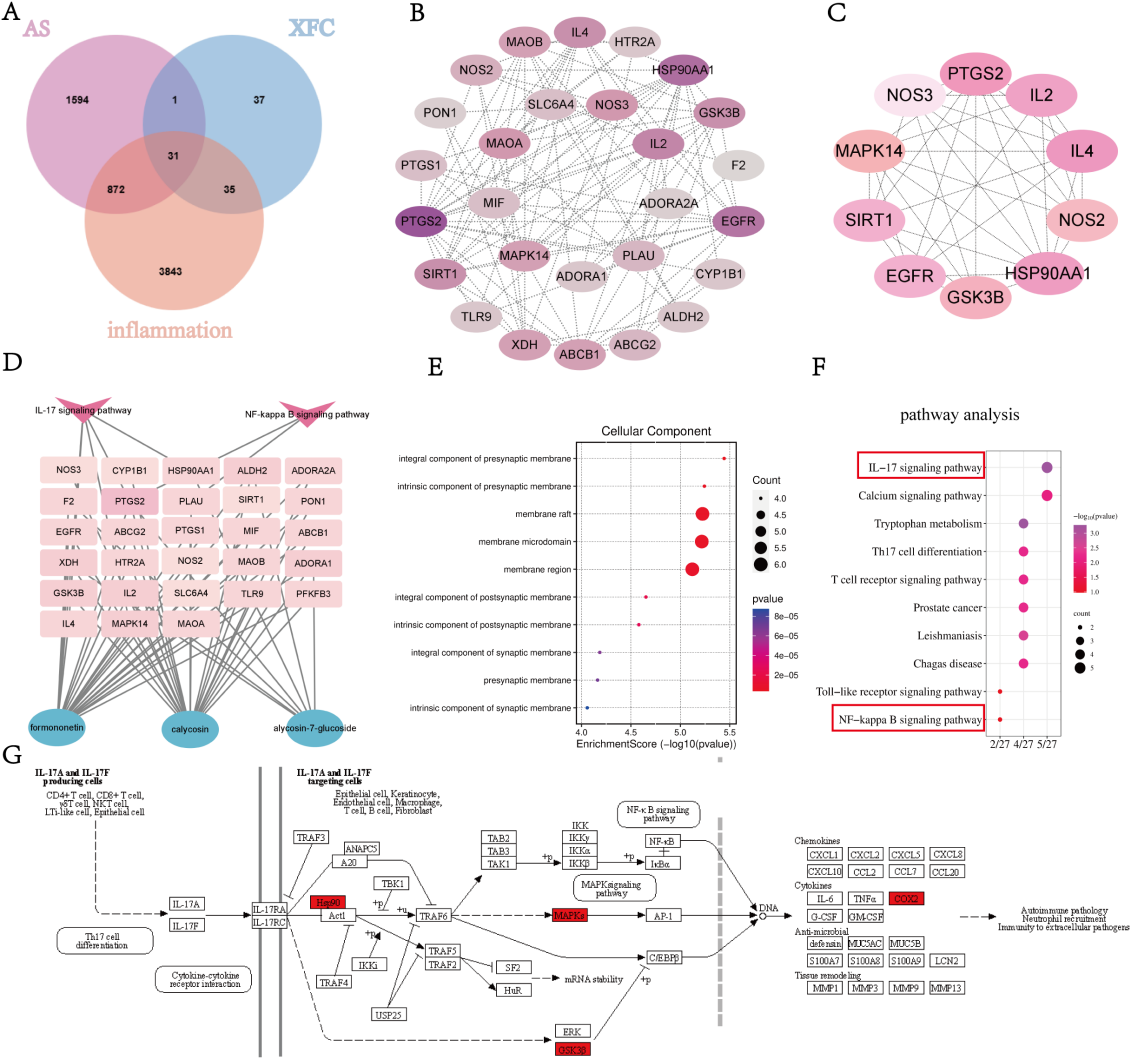
Pharmacology of XFC action on AS inflammation network. **(A)** XFC-AS-inflammation Wayne diagram; **(B)** XFC-AS-inflammation PPI network; **(C)** Identification of the top 10 targets of action based on the hubba plugin; **(D)** XFC component-AS-inflammation-pathway target network diagram; **(E)** CC bioprocesses; **(F)** KEGG analysis; **(G)** IL-17/NF-kB signaling pathway Schematic diagram. Pink squares in D are target genes, red arrows are potential pathways, and green circles are XFC core components.

Based on previous chemical fingerprint analysis of XFC (13), the primary bioactive constituents absorbed into the bloodstream were identified as calycosin glucoside, formononetin, and calycosin (**Supplementary Figures 1**–**4**). The corresponding targets of these compounds were retrieved from the Traditional Chinese Medicine Systems Pharmacology (TCMSP) database (https://tcmspw.com). Disease-related targets associated with AS and inflammation were obtained using the keywords “ankylosing spondylitis” and “inflammation” from multiple databases, including DrugBank, GeneCards, Therapeutic Target Database (TTD), DisGeNET, Online Mendelian Inheritance in Man (OMIM), and PharmGKB. The compound targets of XFC were intersected with AS-related and inflammation-related targets, yielding 31 overlapping targets potentially involved in the therapeutic mechanism of XFC against AS-associated inflammation (**Figure 3A**). These intersecting targets were visualized using Cytoscape software (**Figure 3B**), and an integrated compound–disease–target network was constructed (**Figure 3D**). The CytoHubba plugin was applied to rank the top 10 hub targets within the network (**Figure 3C**). GO and KEGG enrichment analyses were performed on the 31 intersecting genes. GO analysis revealed significant enrichment in cellular component (CC) categories, particularly those related to synaptic membrane conduction (**Figure 3E**). KEGG pathway enrichment identified 183 pathways, among which the IL-17 signaling pathway and the NF-κB pathway were prominently enriched (**Figures 3F, G**). These findings suggest that XFC may exert its therapeutic effects in AS by modulating key immunoinflammatory pathways, particularly the IL-17/NF-κB signaling axis.

### Effects of AS-PBMCs and AS-FLS co-culture on YTHDC1, LINC01579, and inflammatory cytokines

**Figure 4:**
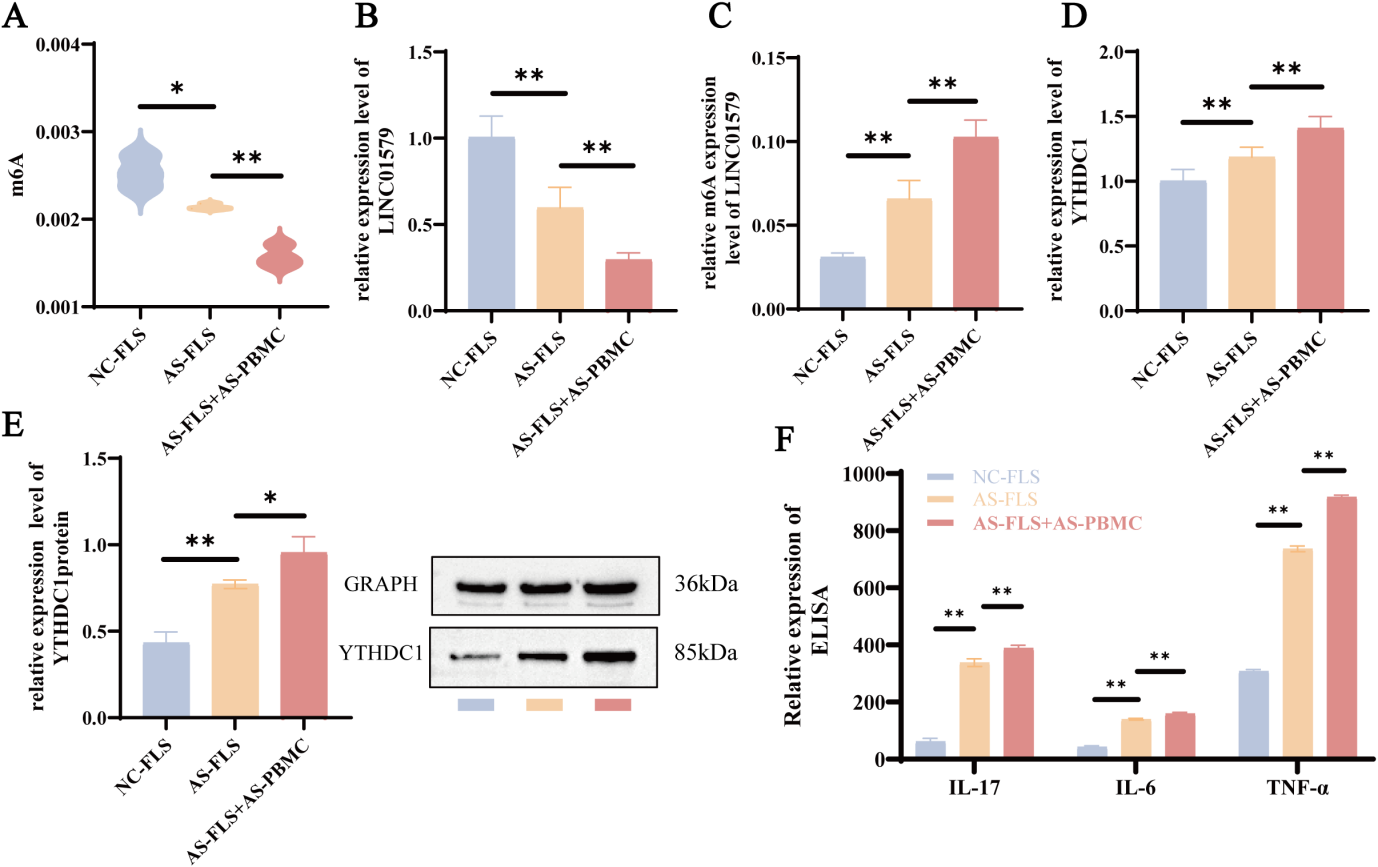
Effects of AS-PBMCs and AS-FLS co-culture model on YTHDC1, LINC01579 and inflammatory cytokines. **(A)** MeRIP-qPCR for total m6A expression, **(B)** RT-qPCR for LINC01579 expression; **(C)** MeRIP-qPCR for LINC0159 m6A expression; **(D)** RT -qPCR to detect the expression of YTHDC1; **(E)** WB to detect the expression of YTHDC1; **(F)** ELISA to detect the expression of IL-6, IL-17 and TNF-a. All experiments were repeated three times. **p<0.01; ***p<0.001.

To better replicate the inflammatory microenvironment of AS *in vitro*, a co-culture system comprising AS-PBMCs and AS-FLS was established. Compared with AS-FLSs cultured alone, the co-culture model exhibited a significantly enhanced inflammatory response, as previously reported (22). MeRIP-qPCR revealed that global m^6^A methylation levels were significantly lower in AS-FLSs relative to normal control FLSs (NC-FLSs), and were further reduced in AS-FLSs co-cultured with AS-PBMCs (*p* < 0.05, *p* < 0.01; **Figure 4A**), consistent with the *in vivo* findings. RT-qPCR analysis showed that LINC01579 expression was significantly decreased in the co-culture group compared with the AS-FLS group (*p* < 0.001; **Figure 4B**). In contrast, the m^6^A modification level of LINC01579 was significantly elevated (*p* < 0.001; **Figure 4C**), indicating enhanced methylation of LINC01579 in the co-culture condition. WB and RT-qPCR analyses further demonstrated that YTHDC1 expression was significantly upregulated in the co-culture model compared with AS-FLSs cultured alone (*p* < 0.001; **Figures 4D, E**). In addition, ELISA results showed markedly increased levels of pro-inflammatory cytokines IL-6, IL-17, and TNF-α in the AS-PBMC-stimulated AS-FLS group (*p* < 0.001; **Figures 4F**), indicating a stronger immune-inflammatory response. Collectively, these findings suggest that the AS-PBMC+AS-FLS co-culture model induces a more robust inflammatory response and altered RNA methylation patterns compared to AS-FLS alone, providing a more representative *in vitro* model for studying AS-related inflammation.

### YTHDC1 regulates LINC01579 stability and inflammatory responses via the MUT1 m6A site

**Figure 5:**
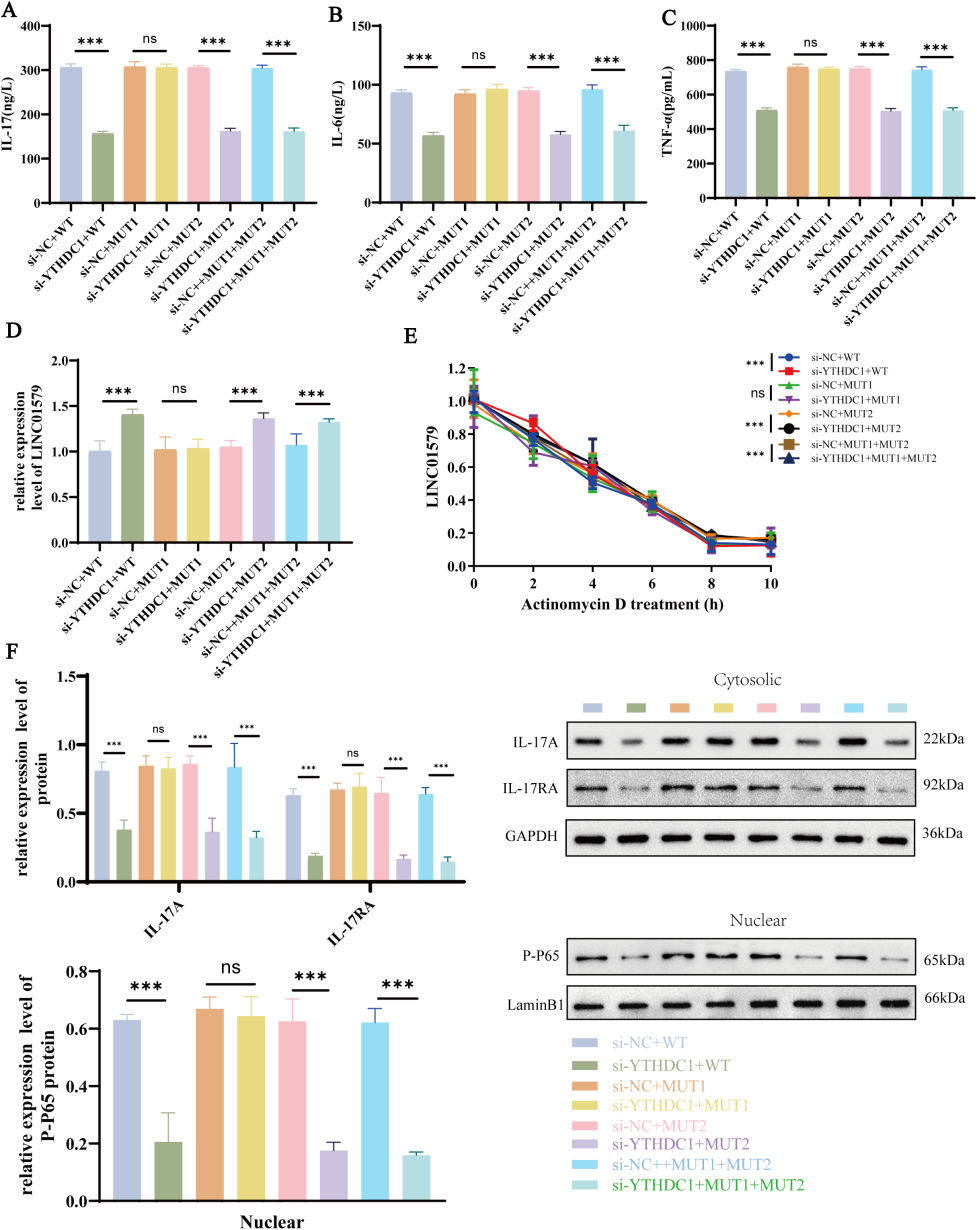
YTHDC1 Regulates LINC01579 Stability and Inflammatory Responses via the MUT1 m6A Site. **(A–C)**. ELISA to detect the expression of IL-6, IL-17 and TNF-a; **(D)** RT -qPCR to detect the expression of LINC0159; **(E)** Radiomycin D assay for LINC0157 stability; **(F)** WB to detect the expression of iIL-17A, IL-17RA, P-P65. All experiments were repeated three times. **p<0.01; ***p<0.001.

To validate the predicted m6A sites on LINC01579, we mutated two candidate sites and examined the effects of YTHDC1 knockdown. In the wild-type (WT) context, YTHDC1 knockdown significantly suppressed IL-17, IL-6, and TNF-α expression (*p* < 0.001, **Figures 5A–C**) while significantly increasing LINC01579 levels (*p* < 0.001, **Figure 5D**). However, mutation of the MUT1 site abolished these effects (*p* > 0.001), identifying MUT1 as the critical functional site. In contrast, mutation of MUT2 alone did not abrogate the regulatory effects (*p* < 0.001), and combined mutation of both MUT1 and MUT2 restored the significant differences upon YTHDC1 knockdown (*p* < 0.001), suggesting a compensatory role for MUT2 in the absence of MUT1. Actinomycin D assays revealed that YTHDC1 knockdown decelerated LINC01579 RNA decay, and this effect was maintained with MUT2 single mutation but restored with combined MUT1+MUT2 mutation, further confirming MUT1 as the key site for YTHDC1-mediated RNA stability (**Figure 5E**). Western blot analysis showed that YTHDC1 knockdown significantly reduced intracellular IL-17A and IL-17RA protein levels, as well as nuclear P-P65 accumulation (*p* < 0.001). These effects were abrogated in the MUT1 mutant context (*p* > 0.001) but remained significant in the MUT2 mutant context (*p* < 0.001, **Figure 5F**). Collectively, these results demonstrate that MUT1 is the primary m6A site through which YTHDC1 regulates LINC01579 and downstream inflammatory responses.

### YTHDC1 regulates IL-17/NF-κB pathway activation via m^6^A modification of LINC01579

**Figure 6:**
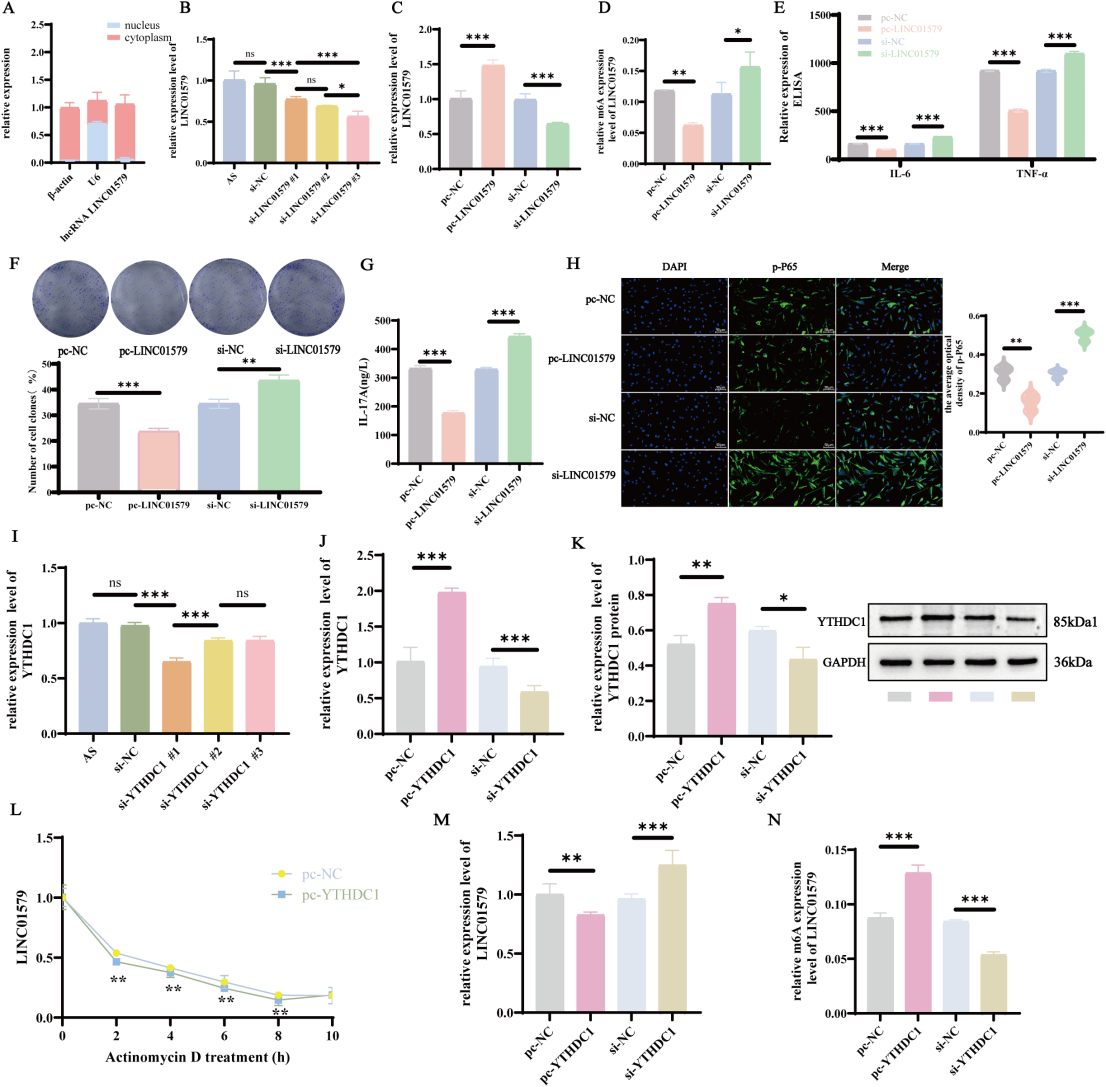
YTHDC1 regulates the activation of IL-17/NF-kB pathway by modulating the expression of LINC0157 m6A. **(A)** Nuclear-cytoplasmic fractionation assay to detect the expression of LINC0157; **(B)** Screening for the optimal small interfering RNA (siRNA) model for LINC0157; **(C)** RT-qPCR to detect the expression of LINC0157; **(D)** MeRIP-qPCR detection of LINC0159 m6A expression; **(E)** ELISA detection of IL-6 and TNF-a expression; **(F)** Colony formation assay to assess colony formation ability; **(G)** ELISA detection of IL-17A expression; **(H)** IF detection of p-P65 expression; **(I)** Screening for the optimal small interfering RNA model for YTHDC1; **(J)** RT-qPCR detection of YTHDC1 expression; **(K)** WB assay for YTHDC1 protein expression. **(L)** Radiomycin D assay for LINC0157 stability. **(M)** RT-qPCR assay for LINC0159 expression, **(N)** MeRIP-qPCR assay for LINC0159 m6A expression. All experiments were repeated three times. *p<0.05; **p<0.01; ***p<0.001.

To elucidate the downstream regulatory mechanisms of LINC01579, we performed gain- and loss-of-function experiments and investigated their effects on relevant signaling pathways. Nuclear-cytoplasmic fractionation analysis revealed that LINC01579 was predominantly localized in the cytoplasm (**Figure 6A**). Among the designed constructs, LINC01579#3 exhibited the highest transfection efficiency (**Figure 6B**). As expected, overexpression of LINC01579 led to an overall reduction in its m6A modification level (possibly due to dilution of the methyltransferase machinery), while knockdown showed the opposite trend (**Figures 6C, D**). consistent with prior *in vivo* observations. Functionally, overexpression of LINC01579 significantly suppressed the expression of pro-inflammatory cytokines, inhibited AS-FLS proliferation, and attenuated IL-17 pathway activation. In contrast, LINC01579 knockdown led to increased inflammatory cytokine expression, enhanced cell proliferation, and activation of the IL-17 pathway (**Figures 6E–G**). IF analysis further revealed that LINC01579 overexpression reduced nuclear translocation of p-p65, while its knockdown promoted p-p65 nuclear accumulation (**Figure 6H**), suggesting that LINC01579 modulates the IL-17/NF-κB pathway by regulating inflammatory responses.

Based on prior bioinformatic predictions, we hypothesized that YTHDC1 is the upstream reader regulating LINC01579. Transfection screening indicated that YTHDC1#1 had the highest knockdown efficiency (**Figure 6I**). Critically, modulating YTHDC1 levels produced the canonical m6A-mediated regulatory effects on LINC01579 (**Figures 6J, K**): YTHDC1 overexpression increased the m6A modification of LINC01579, reduced its RNA stability (**Figure 6L**), and consequently downregulated its mRNA levels. YTHDC1 knockdown had the opposite effects (**Figures 6M, N**). These findings establish that YTHDC1 negatively regulates the stability and expression of the anti-inflammatory lncRNA LINC01579 via m6A modification, thereby modulating the activation of the IL-17/NF-κB pathway.

### XFC reduces YTHDC1 expression, downregulates LINC01579 m^6^A modification, and inhibits NF-κB pathway activation

**Figure 7:**
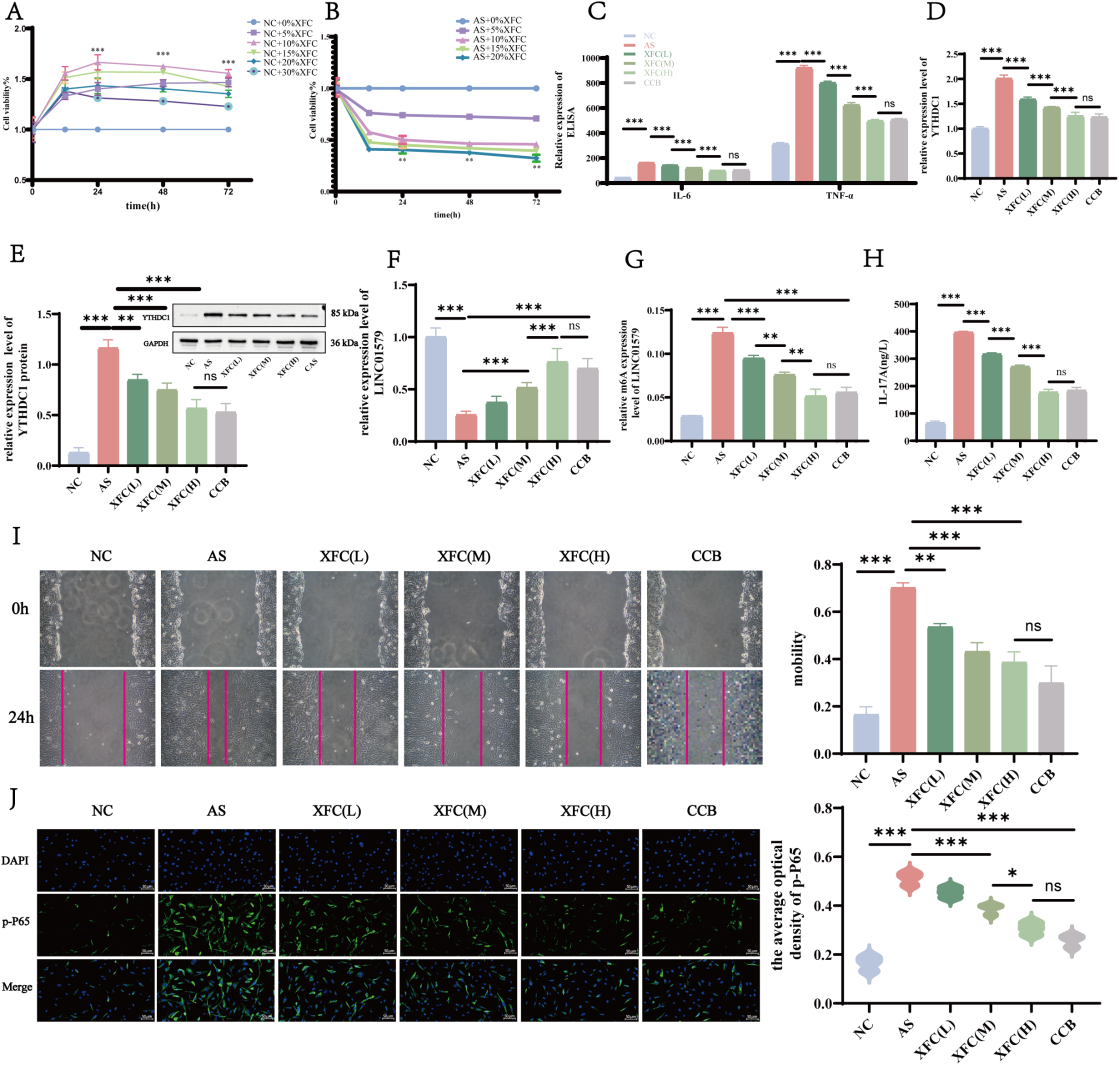
XFC inhibits the level of YTHDC1, raises LINC01579 m6A and downregulates the activation of NF-kB pathway. **(A)** CCK8 assay to measure cell viability of NC-FLS, **(B)** CCK8 assay to measure cell viability of AS-FLS, **(C)** ELISA assay to detect IL-6 and TNF-a expression, **(D)** RT-qPCR assay to detect YTHDC1 expression; **(E)** WB assay for YTHDC1 expression, **(F)** RT-qPCR assay for LINC01579 expression, **(G)** MeRIP-qPCR assay for LINC0159 m6A expression. **(H)** ELISA assay for IL-17A expression, **(I)** Cell scratch assay; **(J)** IF assay for p-P65 expression. All experiments were repeated three times. *p < 0.05; **p < 0.01; ***p < 0.001.

To investigate the regulatory effects of XFC on YTHDC1-mediated m^6^A modification of LINC01579 in an AS cellular model, we first determined non-cytotoxic concentrations of XFC-containing serum using a cell viability assay. CCK-8 results indicated that serum concentrations ranging from 0% to 20% had no significant inhibitory effects on NC-FLS viability at 12, 24, 48, and 72 hours (**Figure 7A**). Based on this, AS-FLSs were subsequently cultured with 10%, 15%, and 20% XFC-containing serum. Among these concentrations, 20% XFC-containing serum demonstrated the most significant inhibitory effect on AS-FLS viability at 48 hours (*p* < 0.001) (**Figure 7B**), and was therefore selected, along with 10% and 15%, for subsequent experiments. CCB treatment served as a control. Following XFC treatment, the expression levels of pro-inflammatory cytokines in AS-FLS were significantly decreased in a concentration-dependent manner (**Figure 7C**). Moreover, XFC significantly downregulated both the mRNA and protein expression of YTHDC1 (**Figures 7D, E**), while upregulating LINC01579 expression (**Figure 7F**) and reducing its m^6^A methylation levels (**Figure 7G**). No significant difference was observed between the high-dose XFC group and the CCB group. Additionally, XFC treatment notably inhibited the activation of the IL-17 pathway (**Figure 7H**). To further evaluate the effect of XFC on cell motility, a wound healing assay was performed. After 24 hours, AS-FLSs treated with XFC or CCB exhibited markedly reduced wound closure rates, indicating impaired migration ability (**Figure 7I**). WB analysis confirmed that XFC treatment significantly reduced p-p65 expression, thereby suppressing activation of the NF-κB pathway (**Figure 7J**). Collectively, these findings suggest that XFC exerts anti-inflammatory effects in AS by suppressing YTHDC1 expression, reducing the m^6^A modification of LINC01579, and inhibiting the activation of the IL-17/NF-κB pathway.

### XFC regulates LINC01579 and downstream inflammatory responses via YTHDC1

**Figure 8:**
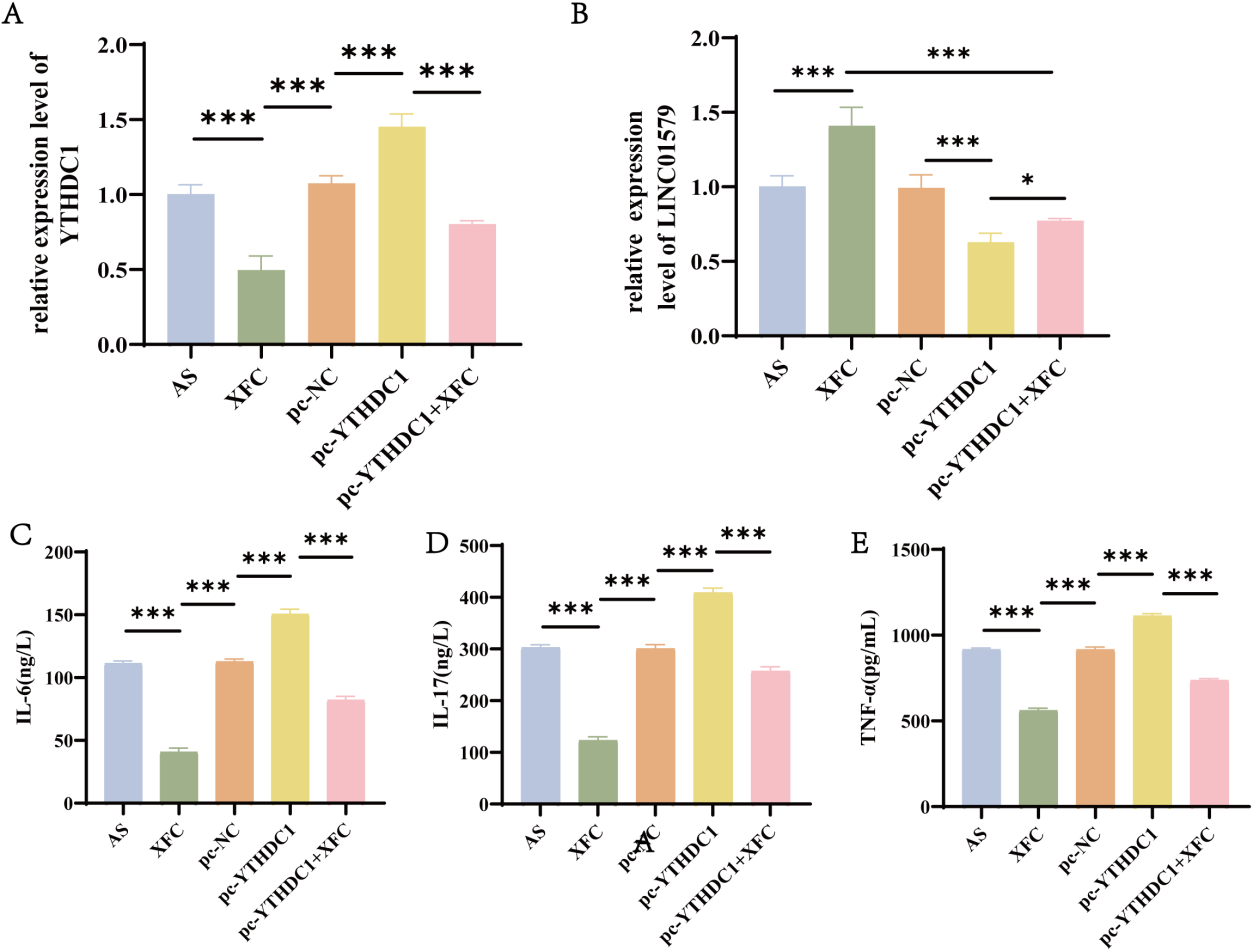
XFC Regulates LINC01579 and Downstream Inflammatory Responses via YTHDC1. **(A)** RT-qPCR assay to detect YTHDC1 expression, **(B)** RT-qPCR assay to detect LINC01579 expression, **(C–E)**. ELISA assay to detect IL-6, IL-17 and TNF-a expression. All experiments were repeated three times. *p < 0.05; **p < 0.01; ***p < 0.001.

To investigate whether XFC regulates downstream LINC01579 through YTHDC1, we performed rescue experiments. Compared with the control group, XFC treatment significantly decreased YTHDC1 expression while increasing LINC01579 levels (**Figures 8A, B**). Overexpression of YTHDC1 significantly elevated both YTHDC1 and LINC01579 levels. Notably, combined treatment with XFC and YTHDC1 overexpression resulted in decreased YTHDC1 and increased LINC01579 compared with YTHDC1 overexpression alone. ELISA assays showed that XFC treatment significantly suppressed the secretion of IL-17, IL-6, and TNF-α, whereas YTHDC1 overexpression significantly promoted these inflammatory cytokines. Importantly, co-treatment with XFC reversed the YTHDC1 overexpression-induced elevation of inflammatory cytokines (**Figures 8C–E**). These results demonstrate that XFC regulates LINC01579 expression and downstream inflammatory responses via YTHDC1.

### XFC ameliorates aberrant RNA methylation and inflammatory responses in PGIA mice

**Figure 9:**
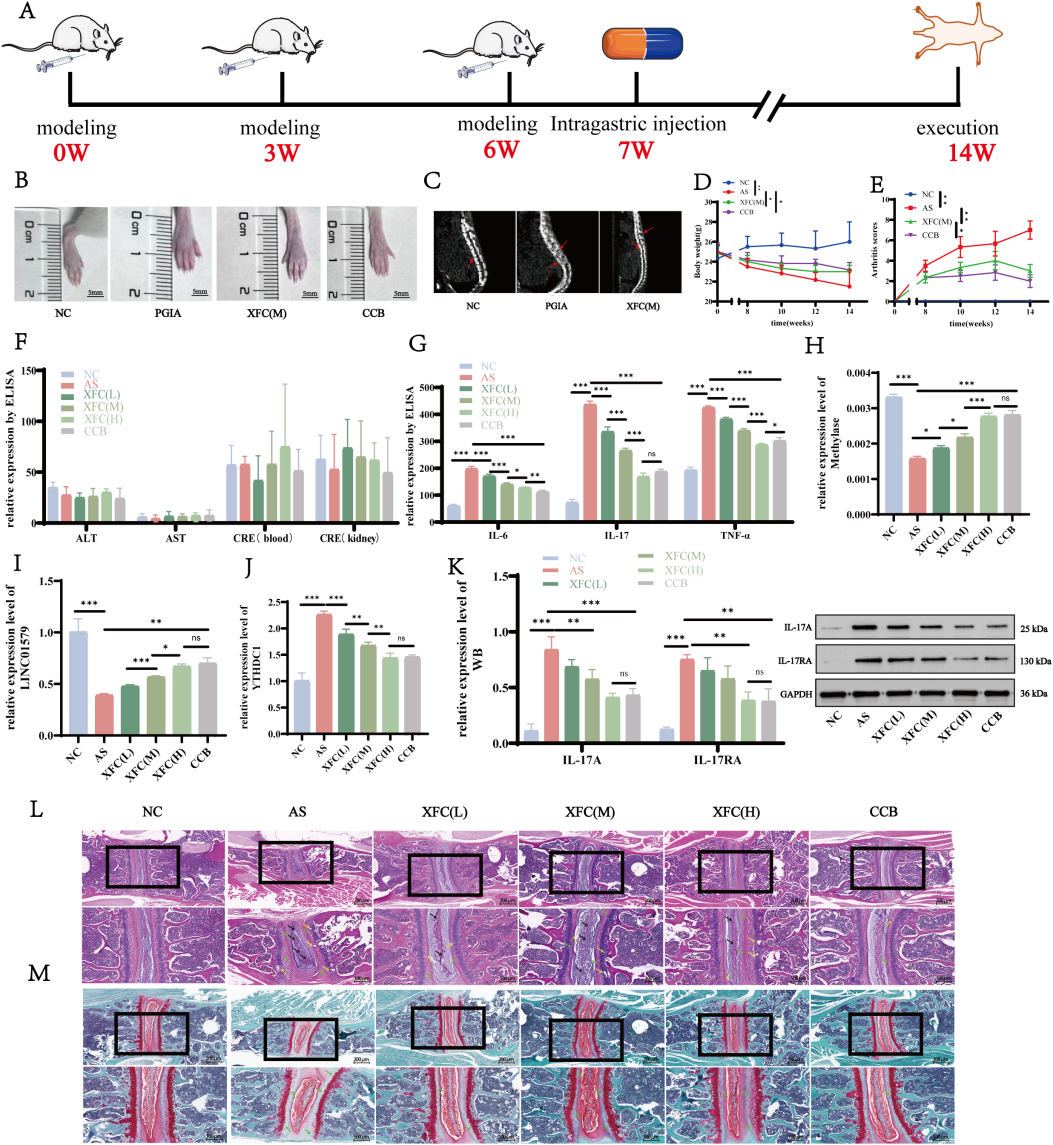
XFC attenuates aberrant methylation and inflammatory response in PGIA mice **(A)** Study design and experimental schedule; **(B)** Representative hind paw observations of four groups of PGIA mice; **(C)** Representative images of Micro-CT scans of spinal joints in three groups, with osteogenesis and stenosis visible in PGIA mice (red arrows); **(D)** Mouse body weight; **(E)** Joint scores; **(F)** ELISA for ALT, AST, CRE (blood and kidney); **(G)**: ELISA for IL-6, IL-17 and TNF-a expression. **(H)** MeRIP-qPCR for total m6A expression. **(I)** RT-qPCR for LINC01579 expression; **(J)** RT- qPCR to detect the expression of YTHDC1; **(K)** WB to detect the expression of IL-17 and IL-17A. **(L)** HE staining of spondyloarthritic joints, PGIA mice were seen with inflammatory cell infiltration and synovial inflammation (black arrowheads), narrowing of joint space, cartilage degradation (green arrows), and bone erosion (yellow arrowheads). **(M)** Saffron O solid green staining of cartilage degradation was seen in PGIA mice (green arrows). All experiments were repeated three times. *p<0.05; **p<0.017

In the PGIA mouse model of AS, the therapeutic effects of XFC on joint pathology and systemic inflammation were evaluated, with CCB used as the positive control. The experimental workflow is illustrated in **Figure 9A**. Following disease induction, PGIA mice exhibited significant paw swelling (**Figure 9B**), an elevated arthritis index (**Figure 9D**), and a decrease in body weight (**Figure 9E**). Micro-CT imaging revealed vertebral joint destruction and inflammatory cell infiltration surrounding the intervertebral discs (**Figure 9C**), confirming successful establishment of the inflammatory arthritis model. Notably, both XFC and CCB treatments alleviated these pathological changes, and no apparent hepatotoxicity or nephrotoxicity was observed during treatment (**Figure 9F**). Serum analysis showed that inflammatory cytokine levels were significantly elevated in PGIA mice (**Figure 9G**), accompanied by reduced global m^6^A RNA methylation levels (**Figure 9H**), decreased LINC01579 expression (**Figure 9I**), and increased YTHDC1 expression (**Figure 9J**). WB analysis further revealed upregulation of IL-17 signaling pathway-related proteins (**Figure 9K**). Treatment with XFC or CCB reversed these changes in a dose-dependent manner, with XFC demonstrating significant efficacy in suppressing pro-inflammatory cytokines, increasing global m^6^A RNA methylation, downregulating YTHDC1, restoring LINC01579 expression, and inhibiting IL-17 pathway activation. Histopathological analysis of spinal joints showed that mice in the PGIA group presented with typical features of spinal joint inflammation, including synovial hyperplasia, inflammatory cell infiltration, cartilage destruction, and bone erosion (**Figures 9L, M**). In contrast, control mice displayed normal histology. Mice treated with XFC or CCB exhibited attenuated inflammation, characterized by reduced inflammatory infiltration and only mild joint space narrowing. Together, these findings demonstrate that XFC effectively improves aberrant m^6^A RNA methylation and inflammatory responses in PGIA mice, consistent with the regulatory effects observed *in vitro*.

### XFC attenuates the inflammatory response in AS by downregulating LINC01579 m^6^A methylation and inhibiting IL-17/NF-κB activation

**Figure 10:**
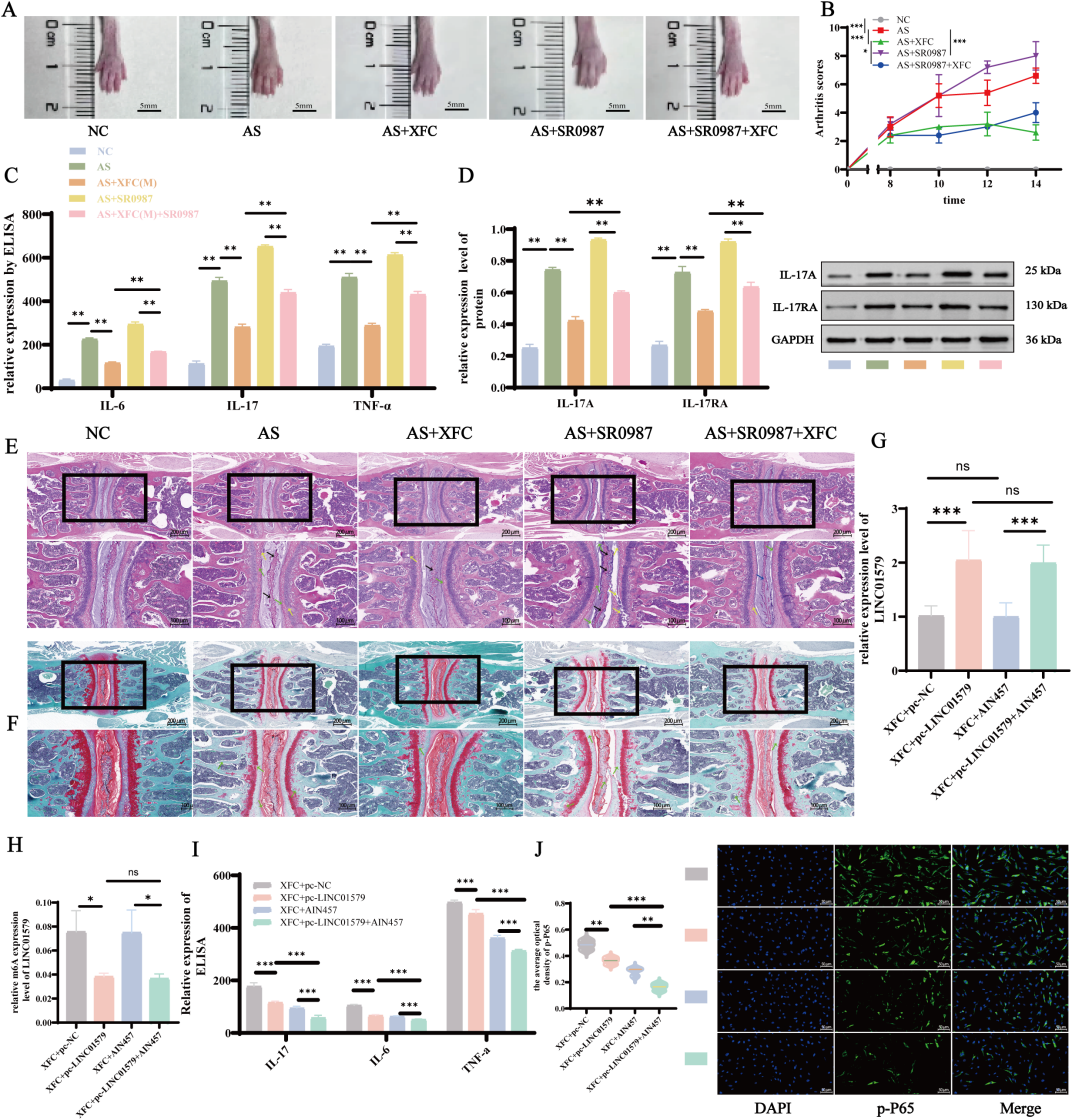
XFC improves AS inflammatory response by upregulating LINC01579 to inhibit IL-17/NF-kB activation **(A)** Representative hindpaws of five groups of PGIA mice; **(B)** Arthritis score; **(C)** ELISA detection of IL-6, IL-17, and TNF-a expression; **(D)** WB detection of IL-17 and IL-17A expression; **(E)** Spinal joint HE staining shows inflammatory cell infiltration and synovial inflammation (black arrows), narrowed joint spaces, cartilage degeneration (green arrows), and bone erosion (yellow arrows) in PGIA mice. **(F)** Fuchsin-O green staining, PGIA mice showed cartilage degeneration (green arrows). **(G)** RT-qPCR detection of LINC01579 expression in AS-FLS; **(H)** MeRIP-qPCR assay for LINC0159 m6A expression; **(I)** ELISA detection of IL-6, IL-17, and TNF-a expression in AS-FLS; **(J)** IF detection of p-P65 expression. All experiments were repeated three times. *p<0.05; **p<0.01.

To determine whether XFC exerts its anti-inflammatory effects through the regulation of LINC01579-mediated m^6^A modification and subsequent inhibition of the IL-17/NF-κB signaling pathway, we conducted a series of *in vivo* and *in vitro* experiments. In the *in vivo* study, an IL-17 agonist, SR0987, was used to perform rescue experiments in PGIA mice. Compared with untreated PGIA controls, administration of SR0987 significantly exacerbated paw swelling and arthritis scores (**Figures 10A, B**), elevated pro-inflammatory cytokine levels (**Figure 10C**), and activated the IL-17 signaling pathway (**Figure 10D**). Co-treatment with XFC effectively reversed the SR0987-induced increases in joint inflammation and pathway activation. Histopathological examination revealed that SR0987 enhanced inflammatory cell infiltration, synovial inflammation, joint space narrowing, cartilage degeneration, and bone erosion (**Figures 10E, F**). These pathological changes were significantly attenuated by XFC co-treatment, indicating that the IL-17/NF-κB pathway plays a key mechanistic role in the therapeutic effects of XFC in AS.

*In vitro*, co-culture experiments were performed using AS-FLSs and AS-PBMCs, with the IL-17 inhibitor Secukinumab (AIN457; Cat# HY-P9927, MCE; 16.2 ng/mL) employed to further elucidate pathway involvement. Following XFC pretreatment, overexpression of LINC01579 reduced its m^6^A methylation level while restoring its transcript expression level. In contrast, AIN457 treatment had no significant effect on LINC01579 expression or m^6^A methylation (**Figures 10G, H**), suggesting that LINC01579 acts upstream of the IL-17 pathway. Both LINC01579 overexpression and AIN457 treatment independently suppressed pro-inflammatory cytokine production and NF-κB pathway activation(**Figures 10I, J**). Notably, their combined use produced a synergistic inhibitory effect, leading to a more pronounced inhibition of inflammatory responses and NF-κB signaling. Collectively, these findings demonstrate that XFC alleviates inflammation in AS by reducing m^6^A methylation of LINC01579, thereby suppressing the activation of the IL-17/NF-κB signaling axis. These results highlight XFC as a promising therapeutic candidate targeting immune-epigenetic crosstalk in AS pathogenesis.

## Discussion

In this study, we demonstrated that YTHDC1 enhanced the IL-17/NF-κB pathway activation by promoting the m^6^A modification of LINC01579. Importantly, we found that XFC effectively inhibited YTHDC1 expression, thereby reducing LINC01579 m^6^A methylation, suppressing the IL-17/NF-κB signaling pathway activation, and alleviating the immune-inflammatory response associated with AS. These findings not only underscore the anti-inflammatory potential of XFC in AS management but also highlight its role in mitigating bone erosion and structural joint damage in a murine model. Compared with other TCM formulations, XFC exhibits a distinct mechanism of action by directly targeting the IL-17/NF-κB axis, which is consistent with current therapeutic strategies for AS. Moreover, its dual regulatory effects, both in suppressing inflammation and potentially promoting bone remodeling or fusion, suggest therapeutic advantages over conventional single-target treatments.

IL-17A plays a pivotal role in the pathogenesis of AS, contributing to immune cell dysregulation and pathological bone remodeling, and has emerged as a key therapeutic target for AS (25). In inflamed target tissues, innate immune cells producing IL-17 are significantly elevated in AS patients (26). Clinically approved IL-17 inhibitors, such as ixekizumab, bimekizumab, brodalumab, and netakimab, have demonstrated therapeutic efficacy in AS. However, their use is frequently associated with adverse events affecting multiple organ systems, including the liver, lungs, skin, gastrointestinal tract, endocrine and nervous systems, and musculoskeletal tissues (27). Moreover, a subset of patients fails to respond or sustain clinical remission, and disease activity may relapse following treatment withdrawal (28, 29). TCM, through its multi-target regulatory properties, has demonstrated promising efficacy in treating immune-mediated disorders, particularly those characterized by chronic joint inflammation and pain. Nonetheless, the complexity of herbal constituents and the lack of clearly defined molecular mechanisms remain major obstacles to its broader clinical integration (30, 31). In this study, we employed an IL-17 pathway agonist *in vivo* and an IL-17 inhibitor *in vitro* to further dissect the mechanism of action of XFC. Our findings from both LINC01579 co-expression assays and *in vivo* rescue experiments confirmed that XFC exerted its therapeutic effects by targeting the IL-17/NF-κB pathway, with LINC01579 acting as a key intermediary regulator in this signaling axis.

Previous studies have demonstrated that DNA methylation modulates the expression of pro- and anti-inflammatory cytokines by altering the methylation status of gene promoter regions (32–34). For example, Xiao et al. (35) conducted an epigenome-wide DNA methylation and transcriptomic analysis of PBMCs from 45 individuals (AS: HC = 30:15) using high-throughput microarrays. A total of 4,144 DEGs were identified. Integrated analysis revealed that an inverse correlation between DNA methylation and gene expression was predominant, with hypermethylation frequently associated with reduced expression. These findings support the concept that DNA methylation functions as a transcriptional “switch” and underscore the significance of epigenetic dysregulation in AS pathogenesis. In addition to DNA methylation, m^6^A modification has emerged as a critical regulatory mechanism in autoimmune diseases, including AS, thereby offering new therapeutic avenues. In this study, XFC was shown to reduce the expression of the m^6^A reader protein YTHDC1 in a dose-dependent manner. YTHDC1 plays a pivotal role in recognizing and binding to specific m^6^A-modified RNA sites (36), subsequently recruiting effector proteins that influence RNA fate, including splicing, nuclear export, stability, and translation (37, 38). Dual-luciferase assays indicated that YTHDC1 bound to two predicted m^6^A modification sites (positions 351/379) within LINC01579, suggesting that YTHDC1 directly regulates the m^6^A status of this transcript. Upon XFC treatment, reduced YTHDC1 levels may diminish its binding to LINC01579, thereby altering its m^6^A modification landscape. RNA stability assays further indicated that YTHDC1 binding enhanced the stability of LINC01579 transcripts, whereas YTHDC1 downregulation decreased LINC01579 m^6^A levels. The resulting stabilization of LINC01579 may prolong its anti-inflammatory effects, thereby attenuating excessive activation of the downstream IL-17/NF-κB pathway. Collectively, these findings suggest that m^6^A modification of LINC01579 modulates inflammatory responses in AS by negatively regulating the IL-17/NF-κB axis. Nevertheless, further in-depth studies are warranted to validate these findings and explore their translational potential in AS treatment.

XFC is a TCM formulation composed of four key herbal ingredients: *Tripterygium wilfordii*, *Astragalus membranaceus*, *Coix lacryma-jobi*, and *Scolopendra subspinipes*. The primary bioactive components of *Astragalus membranaceus* include astragalus polysaccharides and astragaloside, which have been extensively studied for their immunomodulatory and anti-cancer properties (39). Astragalus polysaccharides have been shown to upregulate the expression of various cytokines and chemokines(C. 40) and to inhibit tumor cell migration by suppressing IL-6 and TNF-α production, thereby reducing tumor invasiveness (30). Notably, astragaloside IV and astragalus polysaccharides (APS) have been reported to regulate the Th17/Treg balance, indirectly modulating the IL-17 signaling pathway (41). Coix seed has demonstrated a wide range of pharmacological activities, including inhibition of cell proliferation (42) and attenuation of immune-mediated inflammation (43). Research indicates that coix seed extract can significantly ameliorate paw edema in adjuvant arthritis (AA) rat models and reduce oxidative stress and inflammatory responses (44). *Scolopendra subspinipes* (centipede) has been shown to increase the expression of IL-2, IL-4, and IL-10 in the intestinal tissues of collagen-induced arthritis (CIA) rats. *Tripterygium wilfordii* contains bioactive compounds such as triptolide and tripterygium wilfordii homofuranone. The latter has been identified as a key compound capable of suppressing TNF-α-mediated osteoclastogenesis by inhibiting NF-κB signaling (45). Moreover, it has been reported to inhibit IL-17-induced activation of the NF-κB and MAPK pathways, thereby mitigating inflammatory processes (46). Clinically, XFC has been used for over two decades and has demonstrated substantial efficacy in the treatment of rheumatic and immune-mediated diseases. It has shown therapeutic effects comparable to leflunomide in reducing arthritis-associated biomarkers, while significantly improving patients’ quality of life (47). In the present study, XFC exerted potent anti-inflammatory effects both *in vitro* (AS-FLS/AS-PBMCs co-culture model) and *in vivo* (PGIA mouse model), notably reducing pro-inflammatory cytokine production and ameliorating synovial inflammation. In PGIA mice, XFC treatment markedly improved pathological changes, including reduced bone erosion, joint space narrowing, and cartilage degeneration. Its capacity to inhibit systemic inflammation and limit bone destruction highlights its role in preserving joint integrity and promoting functional recovery. Furthermore, the favorable safety profile observed in our study, together with its multi-targeted mechanism of action, supports the potential of XFC as an effective adjunctive therapeutic strategy for AS management.

Despite these promising findings, this study has several limitations. First, the PGIA mouse model, while useful for studying inflammatory arthritis, does not fully recapitulate the genetic background and male predominance characteristic of human AS. Second, the clinical sample size for protein validation was relatively small, warranting confirmation in larger cohorts. Third, although we provided fingerprint data to ensure XFC batch consistency, the specific active compounds (e.g., calycosin, formononetin) responsible for the observed effects remain to be identified. Fourth, the precise molecular mechanisms by which m6A modification of LINC01579 regulates the IL-17/NF-κB pathway are not fully understood. Future studies should address these limitations by employing more clinically relevant AS animal models, validating findings in multi-center cohorts, systematically identifying the active monomers of XFC, and conducting deeper mechanistic experiments to further elucidate the YTHDC1-LINC01579-IL-17 signaling axis.

## Conclusion

In summary, this study demonstrates, through both *in vitro* and *in vivo* experiments, that XFC effectively attenuates the inflammatory progression of AS by inhibiting the IL-17/NF-κB signaling pathway. This therapeutic effect is mediated via the regulation of YTHDC1-dependent m^6^A modification of LINC01579. These findings highlight the potential of XFC to modulate inflammatory cascades by correcting aberrant m^6^A RNA methylation patterns, thereby offering a novel therapeutic avenue for the clinical management of AS.

## Data Availability

The original contributions presented in the study are included in the article/**Supplementary Material**. Further inquiries can be directed to the corresponding author/s.
