# Supplementary material for: Xinfeng capsule attenuates ankylosing spondylitis by downregulating YTHDC1-mediated m6A modification of LINC01579 and suppressing IL-17/NF-κB signaling
Source: Front Immunol. 2026 Apr 16;17:1762062. doi: 10.3389/fimmu.2026.1762062 (PMC13128365; doi:10.3389/fimmu.2026.1762062)
Supplement: Supplementary file 1 [file DataSheet1.doc]

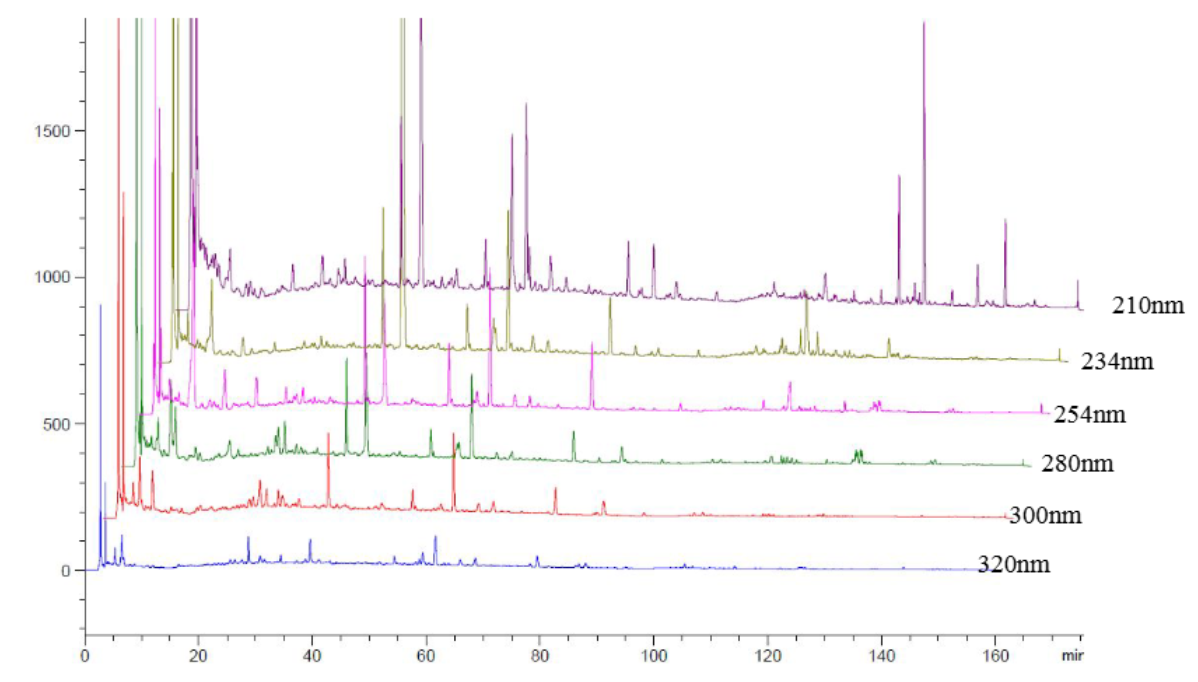


Fig. S1. Xinfeng Capsule HPLC characteristic fingerprint at wavelength of 210 nm、234nm、254 nm、280 nm、300 nm、320 nm.


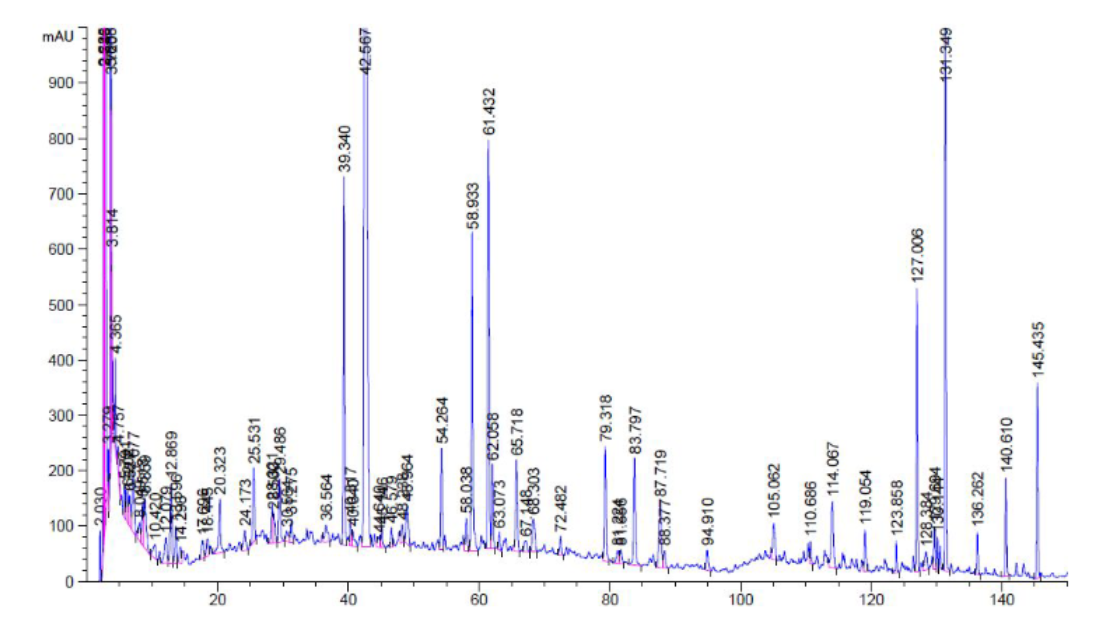


Fig.S2 HPLC characteristic fingerprint of Xinfeng Capsule at 210 nm wavelength.


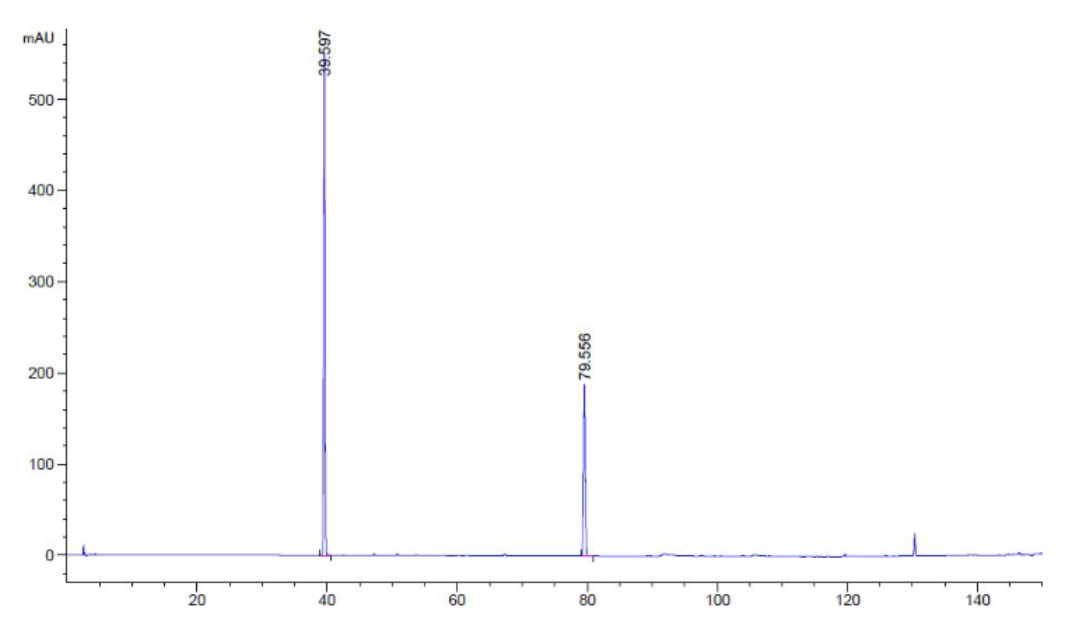


Fig.S3 Calycosin glucoside, formononetin mixed reference HPLC chromatogram of 210 nmwavelength.


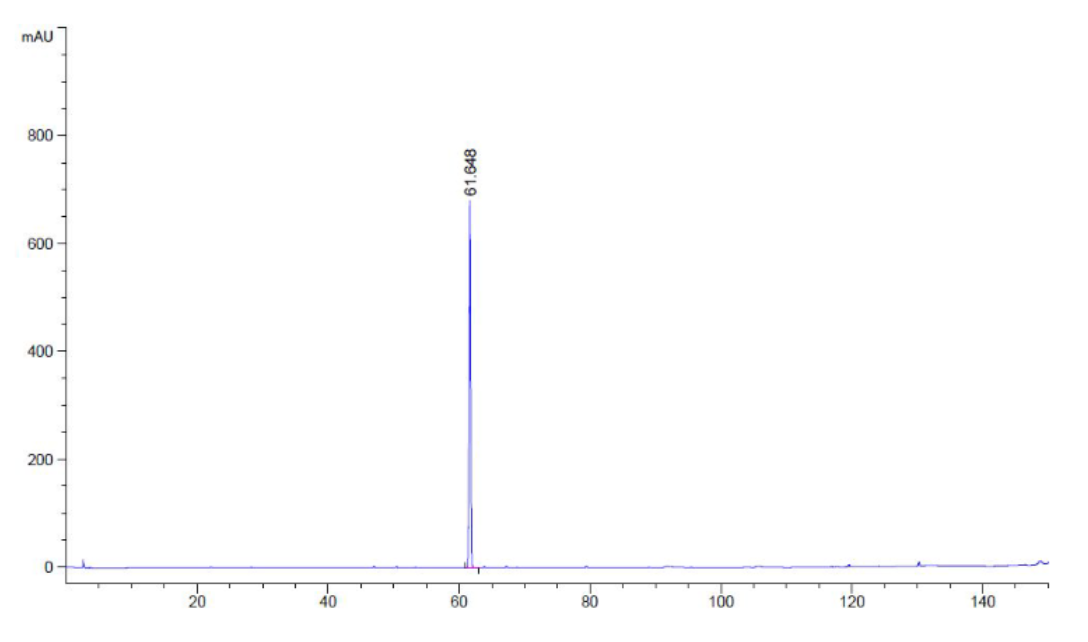


Fig. S4 Calycosin control HPLC chromatogram of 210 nm wavelength
